# 1,4,5,8-Naphthalenetetracarboxylic dianhydride grafted phthalocyanine macromolecules as an anode material for lithium ion batteries

**DOI:** 10.1039/d1na00115a

**Published:** 2021-03-27

**Authors:** Lihong Tao, Jianjun Zhao, Jun Chen, Caixia Ou, Weixia Lv, Shengwen Zhong

**Affiliations:** School of Materials Science and Engineering, Jiangxi Provincial Key Laboratory of Power Batteries and Materials, Jiangxi University of Sciences and Technology Ganzhou 341000 China chenjun@jxust.edu.cn zhongshw@126.com

## Abstract

For solving the problems of high solubility in electrolytes, poor conductivity and low active site utilization of organic electrode materials, in this work, 1,4,5,8-naphthalenetetracarboxylic dianhydride (NTCDA) grafted nickel phthalocyanine (TNTCDA-NiPc) was synthesized and used as an anode material for lithium ion batteries. As a result, the dispersibility, conductivity and dissolution stability are improved, which is conducive to enhancing the performance of batteries. The initial discharge capacity of the TNTCDA-NiPc electrode is 859.8 mA h g^−1^ at 2 A g^−1^ current density, which is much higher than that of the NTCDA electrode (247.4 mA h g^−1^). After 379 cycles, the discharge capacity of the TNTCDA-NiPc electrode is 1162.9 mA h g^−1^, and the capacity retention rate is 135.3%, which is 7 times that of the NTCDA electrode. After NTCDA is grafted to the phthalocyanine macrocyclic system, the dissolution of the NTCDA in the electrolyte is reduced, and the conductivity and dispersion of the NTCDA and phthalocyanine ring are also improved, so that more active sites of super lithium intercalation from NTCDA and phthalocyanine rings are exposed, which results in better electrochemical performance. The strategy of grafting small molecular active compounds into macrocyclic conjugated systems used in this work can provide new ideas for the development of high performance organic electrode materials.

## Abstract 2

A large-volume branched phthalocyanine conjugated TNTCDA-NiPc that is electrolyte-stable with many active sites was synthesized. The capacity, cycling stability, rate capability of TNTCDA-NiPc electrode are greatly improved compared with NTCDA electrode.

## Graphical abstract

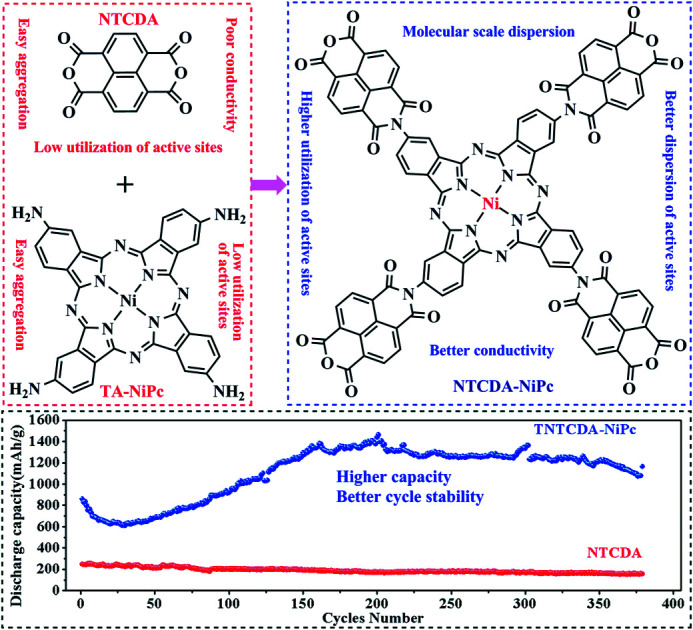

## 1 Introduction

At present, with the increasing demand for energy storage equipment, large capacity, a long life and low cost have become the target of lithium ion batteries in the future.^1,2^ In lithium ion batteries, the electrode material is the key factor to determine the performance of the battery. Nowadays, traditional carbon negative materials which have been commercialized have some problems such as limited theoretical capacity, unstable layered structures and poor cycling stability and so on, which limit their further wide application.^3–5^ Therefore, it is urgent to develop new anode materials with high performance.^6,7^ Because of its advantages of high theoretical specific capacity, abundant raw materials, environmental friendliness, strong structural design and safe systems, organic compounds,^8–13^ as negative electrode materials of lithium ion batteries, meet the requirements of being green, safe and efficient, and provide new possibilities and prospects for the development of clean and efficient lithium ion batteries. Among them, oxygen-containing active compounds (carbonyl, quinone, nitro, *etc.*) have attracted much attention as a class of emerging electrochemical energy storage materials.^14–20^

Although small molecules of oxygen-containing conjugated organic anode materials can obtain high discharge capacity,^17,21^ most of them show poor cycling performance and rate performance due to the following factors.^22–24^ Firstly, organic electrode substances (especially organic materials with a small molecular weight) may be dissolved in electrolyte during cycling, resulting in poor cycle stability; secondly, small molecules are easily aggregated to limit the exposed active sites, resulting in low capacity and coulomb efficiency; Thirdly, organic electrode materials usually show low conductivity, resulting in poor rate performance. In response to these problems, researchers conducted a series of studies and proposed many solutions, such as polymerizing small molecules to improve solubility, and adding a large number of conductive agents^25–27^ or composites with high conductive materials^28–30^ to improve the conductivity. These strategies and methods provide a good idea and reference for improving the properties of organic electrode materials. However, these methods also bring many new problems, such as the polymerization method can reduce the solubility, but cannot improve the conductivity; the method of adding conductive agents can improve the conductivity but can cause the decrease of the effective capacity of the electrode. Therefore, the design of a new organic electrode material can not only solve the problems of high solubility, poor conductivity and limited active point, but also does not result in new negative factors that cause performance decline, and has become the focus of future research on organic electrode materials.

Herein, in this work, NTCDA grafted nickel phthalocyanine (TNTCDA-NiPc) was synthesized and used as an anode material for lithium ion batteries. As a result, dispersibility, conductivity and dissolution stability are greatly improved, which results in excellent performance of lithium ion batteries. The strategy of grafting small molecular active compounds into macrocyclic conjugated systems used in this work can provide new ideas for the development of high performance organic electrode materials.

## 2 Experimental

### 2.1 Materials

All materials used in this experiment, such as 4-nitrophthalic anhydride, 1,4,5,8-naphthalenetetracarboxylic dianhydride (NTCDA), nickel chloride, *n*-pentanol, *N*,*N*-dimethylformamide (DMF), sodium sulfide nonahydrate, super P, anhydrous ethanol, poly(vinylidene fluoride) (PVDF), *N*-methylpyrrolidone (NMP) and so on, are purchased from Aladdin Chemical Reagent Co., Ltd, and are used directly without any purification.

### 2.2 Synthesis

#### 2.2.1 Tetra-β-(4-nitro) nickel phthalocyanine (TN-NiPc)

A mixture of 4-nitrophthalic anhydride (0.15 mol, 25.9596 g) and *n*-pentanol (300 mL) was stirred at 60 °C for 30 minutes under a nitrogen atmosphere. After that, the mixture was heated up to 80 °C, and 5 or 6 drops of 1,8-diazabicyclo[5.4.0]undec-7-ene (DBU) was added into the mixture as the catalyst, then the mixture was heated up to 135 °C, and the dried nickel chloride (4.9 g, slightly excessive) was added and refluxed for another 65 hours. After the reactant was cooled to room temperature, anhydrous ethanol (300 mL) was added to the mixture and allowed to rest overnight. The green crude product was precipitated, filtered, then washed repeatedly with anhydrous ethanol, and vacuum dried to obtain blue-green compound TN-NiPc.

#### 2.2.2 Tetra-β-(4-amino) nickel phthalocyanine (TN-NiPc)

A mixture of TN-NiPc (0.1 mol, 7.6 g), and Na_2_S·9H_2_O (0.12 mol, 28.8 g) were added to DMF (300 mL) after re-steaming, stirred at room temperature until the mixture was dissolved completely, and then heated up to 65 °C to react for 1 hour to obtain a dark green liquid. After the reactant was cooled to room temperature, deionized water (300 mL) was added to the mixture, stirred to precipitate solid, and allowed to rest overnight; the mixture was washed repeatedly with deionized water until the filtrate was clear and transparent; then the mixture was filtered and washed with anhydrous ethanol until the filtrate was clear and transparent and vacuum dried overnight, to obtain a dark green solid compound TA-NiPc.

#### 2.2.3 Tetra-β-1,4,5,8-NTCDA substituted nickel phthalocyanine (TNTCDA-NiPc)

TA-NiPc (0.25 g, 0.4 mmol), and NTCDA (0.45 g, 1.68 mmol) were dissolved in re-distilled DMF, respectively, to obtain solution L_1_ and solution L_2_. Then L_1_ was added into L_2_, and the mixture was heated to 70 °C slowly; after these two solutions were mixed completely, they continued to react at 70 °C overnight to obtain a dark green liquid. After the reaction mixture was cooled to room temperature, it was filtered and washed with distilled water repeatedly and dried in a vacuum at 80 °C overnight, to obtain a dark green compound TNTCDA-NiPc.

### 2.3 Characterization and electrochemical tests

UV-Vis spectroscopy and dissolution experiments were carried out on a Hitachi U-3010 spectrophotometer. The infrared spectrum was obtained with a Fourier transform infrared (FTIR) spectrophotometer Bruker IFS66/S. X-ray diffraction spectroscopy (XRD) was conducted with a X-ray diffractometer of Cu Kα radiation. The scanning electron microscopy (SEM) image was measured on a ZEISS Crossbeam 340 scanning electron microscopy analyzer. X-ray photoelectron spectroscopy (XPS) was captured on a Thermo Scientific K-Alpha.

Before the electrochemical performance test, all cells were pretreated by discharging to 0.01 V from the open-circuit voltage (OCV) at a 200 mA g^−1^ current density and eventually charged to 3.0 V at the same current density. Through the Arbin Battery Test Station BT2000, Arbin Instruments, College station (TX, USA) the electrochemical performance was evaluated. A Solatron 1260/1287 Electrochemical interface (Solatron Metrology, Bognor Regis, UK) was used for cyclic voltammetry tests at a scan rate of 0.2 mV s^−1^ between 0.01 and 3.0 V. Impedance analysis was conducted using a Shanghai Chenhua Chi660e.

### 2.4 Preparation of electrode sheets and batteries

TNTCDA-NiPc powder, super P and poly(vinylidene fluoride) (PVDF) binder were mixed at a mass ratio of 6 : 3 : 1, then 1 g of this mixture was added to 6 mL of *N*-methylpyrrolidone (NMP) and ball-milled until the slurry became uniform. The uniform slurry was coated on copper foil uniformly, and dried at 120 °C for an hour, finally vacuum drying at 60 °C for 24 h. The film was cut into a circular piece of 1.2 cm diameter; the actual loading amount of the active material was 0.996 mg and the area mass loading of the electrode was calculated to be 0.88 mg cm^−2^. Then Li-ion batteries are assembled with lithium metal sheets as counter electrodes, and 1 M LiPF_6_ in a mixture of ethylene carbonate/diethyl carbonate/ethyl methyl carbonate (EC/DEC/EMC, 1 : 1 : 1 by volume) as the electrolyte (purchased from Capchem Technology (Shenzhen) Co., Ltd).

## 3 Results and discussion

**Fig. 1:**
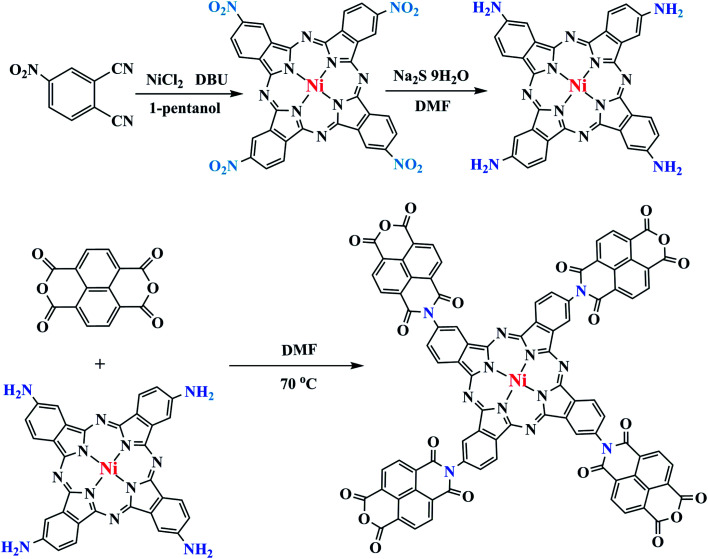
Synthesis process of TNTCDA-NiPc.

NTCDA was grafted to TA-NiPc through a series of reactions (Fig. 1). Firstly, the cyclic reaction of 4-nitrophthalic anhydride and dried NiCl_2_ occurred to produce tetra-β-(4-nitro) nickel phthalocyanine (TN-NiPc) under the action of DBU as a catalyst. Then tetra-β-(4-amino) nickel phthalocyanine (TA-NiPc) was obtained by reducing the nitro group in TN-NiPc. Lastly, NTCDA reacted with the amino groups on the TA-NiPc ring to obtain the corresponding phthalocyanine TNTCDA-NiPc. In this work, replacing NTCDA with phthalocyanine rings will produce the following effects. On the one hand, the dispersion of NTCDA and phthalocyanine rings can be improved from the molecular point of view, which can effectively increase the number of active points of C=C and C=O bonds. On the other hand, the stability of NTCDA in electrolyte solvent is improved. Finally, it will be very beneficial to the electrochemical performance of active substances.

**Fig. 2:**
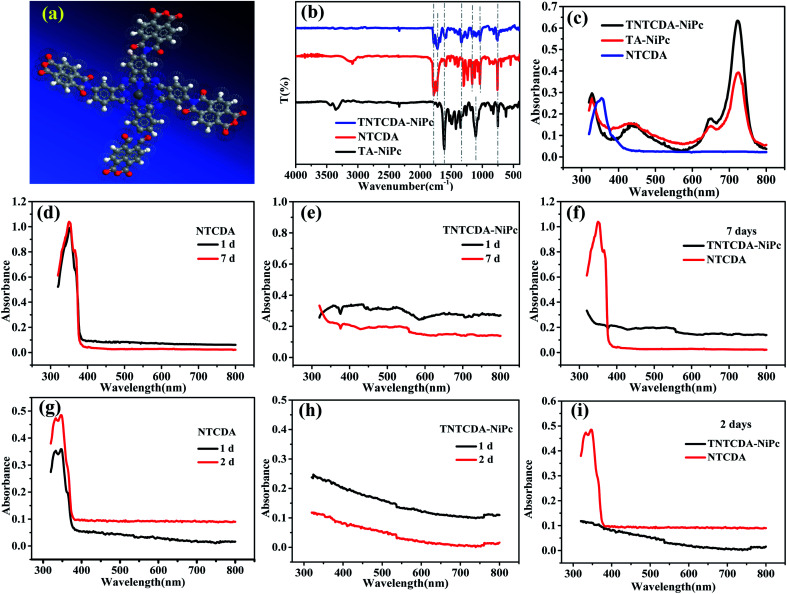
(a) TNTCDA-NiPc molecular structure; (b) FTIR spectra of TNTCDA-NiPc powder; (c) UV-Vis spectra of TNTCDA-NiPc (5 × 10^−6^ M), TA-NiPc (5 × 10^−6^ M) and NTCDA (4 × 5 × 10^−6^ M) in DMF; (d–f) UV-Vis spectra of TNTCDA-NiPc and NTCDA in DMC/EMC/EC = 5 : 3 : 1 at different times (1 × 10^−4^ M); (g–i) UV-Vis spectra of TNTCDA-NiPc and NTCDA in DMC/EMC/EC = 1 : 1 : 1 at different times (1 × 10^−4^ M).

Fig. 2a shows the optimized molecular structure diagram of TNTCDA-NiPc. After grafting, the four NTCDA were distributed around the phthalocyanine ring, which separated them effectively from the molecular point of view and increased the active point. Fourier transform infrared (FTIR) spectra were measured as shown in Fig. 2b. For the NTCDA sample, the peaks at 3086 cm^−1^ are attributed to the tensile vibration of the =C–H of benzene ring, and the peaks at 1782, 1729 and 1162 cm^−1^ are attributed to the tensile vibration of the C=O and C–O bonds, respectively. The peak at 1038 cm^−1^ is the tensile vibration of the C–O–C bond on the phthalocyanine ring, and the peaks at 756 cm^−1^ are attributed to the –Ar–H structure. And for the TA-NiPc sample, the peaks at 3447 and 3356 cm^−1^ are attributed to the tensile vibration of the –N–H of phthalocyanine, and the peaks at 1616 and 1351 cm^−1^ are attributed to the tensile vibration of the C=N and C–N bonds on the Pc conjugated structure, respectively. The peaks at 752 and 1102 cm^−1^ are attributed to the Pc-ring structure and the bending vibration of the C–H bond on the Pc ring, respectively. After the NTCDA substituted onto the TA-NiPc, for the TNTCDA-NiPc compound, the peaks at 1781, 1722 and 1163 cm^−1^ are attributed to the tensile vibration of the C=O and C–O bonds of grafted NTCDA, respectively, and the peak at 1039 cm^−1^ is attributed to the tensile vibration of the C–O–C bond on the phthalocyanine ring. The peaks at 1607 and 1334 cm^−1^ are attributed to the tensile vibration of the C=N and C–N bonds on the Pc conjugated structure, respectively, and the peaks at 755 and 1039 cm^−1^ are attributed to the Pc-ring structure and the bending vibration of the C–H bond on the phthalocyanine ring, respectively, and the peaks at 755 are attributed to the Pc-ring structure. The results of FTIR indicated that the target product TNTCDA-NiPc was successfully synthesized. According to the UV-Vis characteristic absorption (Fig. 2c and Table 1), it can be seen that the UV-Vis characteristic absorption of NTCDA only presents a single peak at 352 nm, while after NTCDA was substituted to the Pc ring, TNTCDA-NiPc shows obvious characteristic absorption peaks of Pc compounds,^31^ including a broad B band at 328 nm, shoulder peaks at 650 nm, and the strongest Q band at 724 nm. The absorption peaks of the two Pcs are the same basically without deviation, but the absorption peaks of TNTCDA-NiPc at 328, 650 and 724 nm are higher than those of TA-NiPc, especially at 724 nm, indicating that its aggregation effect is smaller.

**Table 1:** UV-Vis absorption parameters of TNTCDA-NiPc, TA-NiPc and NTCDA in DMF

Sample	B band		Q band	
TNTCDA-NiPc	328 (0.296)	432 (0.152)	650 (0.179)	724 (0.634)
TA-NiPc	328 (0.272)	434 (0.157)	650 (0.144)	724 (0.393)
NTCDA	354 (0.274)	—	—	—

To explore the solubility changes after NTCDA substituted on the Pc ring, the TNTCDA-NiPc and NTCDA compounds were added into an electrolyte solution (DMC : EMC : EC = 5 : 3 : 1), respectively, which can reach a concentration of 10^−4^ mol L^−1^; the UV-Vis characteristic absorption spectra (Fig. 2d–f) of the solution were compared at different standing times. Its obvious that NTCDA molecules (Fig. 2d) in the electrolyte solvent has a high solubility. The absorbance at 350 nm increased from 0.99 to 1.04 with the time of placement from 1 day to 7 days, indicating that the dissolution of NTCDA in the electrolyte increased gradually with the increase of placement time. However, the TNTCDA-NiPc compound (Fig. 2e) is almost insoluble in the electrolyte solvent, whether placed for 1 day or more than 7 days, and the obvious absorbance in the UV-Vis curves (Fig. 2f) cannot be seen, which fully indicates that the solubility in the electrolyte is obviously inhibited after NTCDA is replaced on the phthalocyanine ring, which will be very beneficial to the stability of the battery. To verify whether the proportion of electrolyte solvents will cause the difference in solubility, dissolution experiments with exactly the same electrolyte (DMC : EMC : EC = 1 : 1 : 1) in the battery were performed and their UV-Vis absorption spectra were tested, as shown in Fig. 2g–i. Obviously, no matter what the proportion of electrolyte solution is, NTCDA sample has obvious characteristic absorption peak of Q band and B band, but for the TNTCDA-NiPc sample, no characteristic absorption peak is observed. The results show that the solvent ratio of electrolyte has no obvious effect on solubility and will not change the conclusion.

**Fig. 3:**
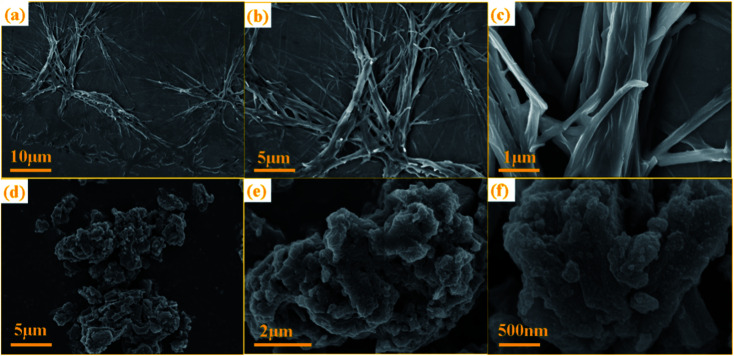
SEM images of NTCDA powders (a–c) and TNTCDA-NiPc powders (d–f).

The SEM images of NTCDA and TNTCDA-NiPc were investigated as shown in Fig. 3; it can be found that there are obvious differences in their morphologies. NTCDA is a fiber or polyfiber with a diameter of about 100 nm, with a smooth and flat surface, whose structure is dense and the specific surface area is small, which is not conducive to the transport of Li-ions. However the TNTCDA-NiPc sample has a loose porous structure with the size of the primary particles being about 100 nm and secondary particles accumulated by primary particles being about 5 μm. The result indicates that after NTCDA grafted onto Pc rings, the microscopic morphology changed greatly; the obtained TNTCDA-NiPc presents a loose porous structure with a larger specific surface area, which is more favorable for lithium ion transport and exposure to more active sites.

**Fig. 4:**
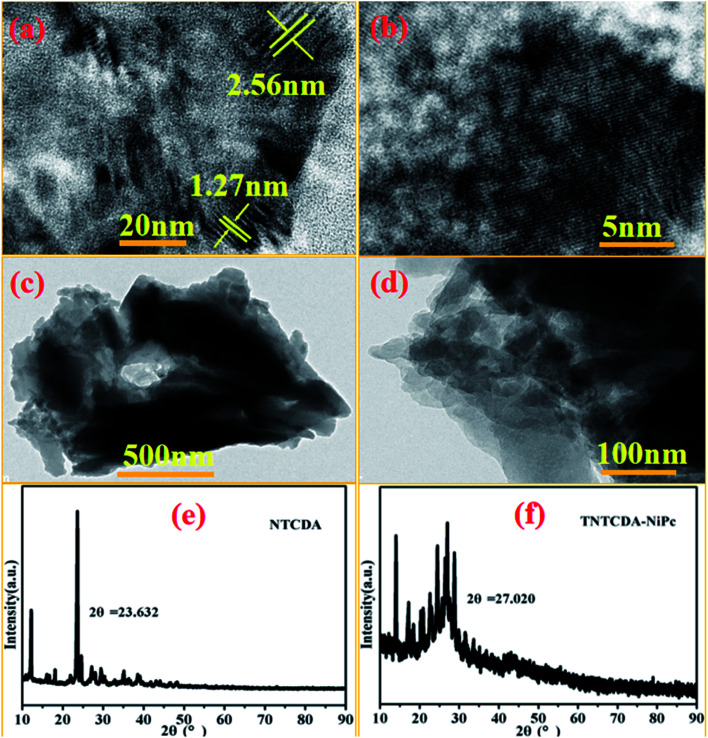
TEM images of NTCDA (a and b) and TNTCDA-NiPc (c and d) with ethanol as a dispersant; (e and f) XRD diffraction patterns of NTCDA and TNTCDA-NiPc powders.

Combined with the TEM images as shown in Fig. 4, NTCDA has obvious lattice fringes with the size of 1.27 nm and 2.56 nm in different directions (Fig. 4a and b), while TNTCDA-NiPc has no distinct layered structure, and an amorphous morphology can only be observed. The absence of obvious lattice fringes indicates low crystallinity, which is consistent with its XRD diffraction pattern as shown in Fig. 4e and f. NTCDA has obvious diffraction peaks at 23.63° with the calculated interlayer spacing of 3.76 Å while TNTCDA-NiPc has no obvious diffraction peak in the XRD diffraction pattern’ at 27.02° there is a slightly obvious wide peak corresponding to the interlayer spacing, which can be calculated using the Bragg equation (3.30 Å). This amorphous structure is more beneficial to the transport of lithium ions.

**Fig. 5:**
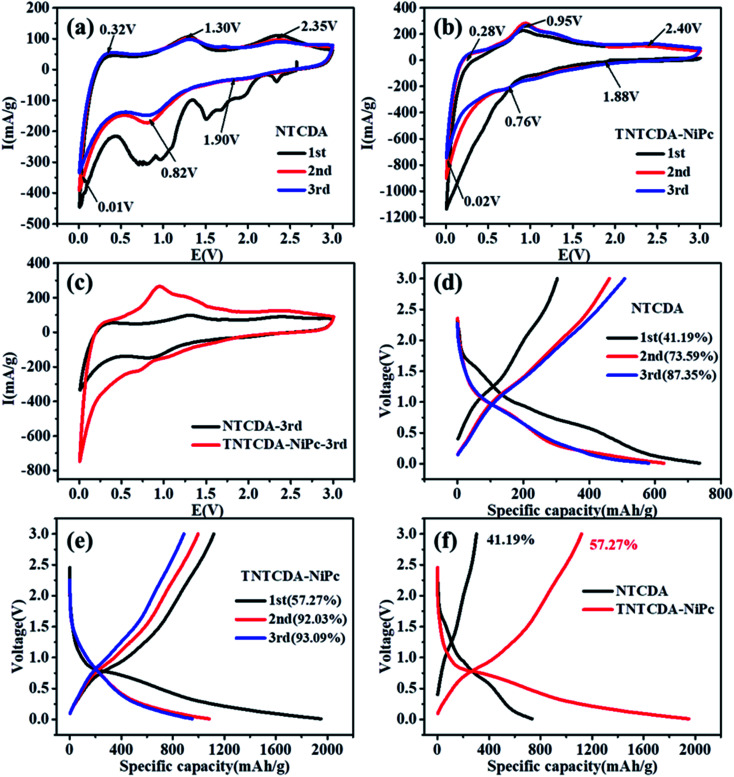
Cyclic voltammetry curves of the (a) NTCDA electrode; (b) TNTCDA-NiPc electrode; (c) contraction of the third CV curves; (d) charge/discharge curves of the NTCDA electrode; (e) charge/discharge curves of the TNTCDA-NiPc electrode; (f) contraction of initial charge–discharge curves of both electrodes (200 mA g^−1^).

After the NTCDA and TNTCDA-NiPc electrodes were prepared and assembled into a Li-ion battery. The cyclic voltammogram (CV) test was carried out from 0.01 to 3.0 V with a scanning rate of 0.2 mV s^−1^. Fig. 5a shows that the CV curves of the NTCDA electrode are similar to those of the traditional layered anode material,^32^; three pairs of redox peaks at 2.35/1.90 V, 1.30/0.82 V and 0.32/0.01 V were observed, which correspond to the lithium intercalation reaction of C=O and C=C functional groups in NTCDA and the interlayer, respectively. The CV curve of the first circle shows the most obvious redox peak and the largest closed area, which indicates that the NTCDA electrode has a large irreversible capacity loss after initial charging and discharging. However for the electrode of TNTCDA-NiPc (Fig. 5b), three pairs of redox peaks, at 2.4/1.88 V, 0.95/0.76 V and 0.28/0.02 V were observed, which correspond to the lithium intercalation reaction of the peripheral NTCDA group and phthalocyanine macrocyclic conjugate structure, as well as interlayer spacing. The closed area of the first CV curve of the TNTCDA-NiPc electrode is slightly larger than that of the last two times, which indicates that it has a smaller irreversible capacity, but the high coincidence degree of the redox peak of the CV curves indicates that it has better reversibility. Comparing the third CV curves of the two electrodes (Fig. 5c), the closed area of CV curves for the TNTCDA-NiPc electrode was greatly larger than that of the NTCDA electrode, indicating it has a higher specific capacity.

Fig. 5d shows the initial three charge/discharge curves of the NTCDA electrode, and a low initial coulombic efficiency of 41.2% is observed. For the next two cycles, the coulomb efficiency was only 73.6% and 87.4%. However for the TNTCDA-NiPc electrode (Fig. 5e), the first three coulombic efficiencies were 52.3%, 92.0% and 93.1%, respectively, which were much higher than those of the NTCDA electrode. The irreversible capacity loss in the early stage of the NTCDA and TNTCDA-NiPc electrodes is due to the following reasons. Firstly, the electrode with a low potential reacts with the electrolyte for the first time to form a passivation film (SEI film) with lithium ion conductivity and electronic insulation, and its irreversible capacity is positively related to the surface area of electrode materials wetted by electrolyte. In this work, the NTCDA and TNTCDA-NiPc electrodes contain C=O, C=N and C=C unsaturated double bonds, which have good compatibility with the electrolyte of carbonate composition. During the static process after the lithium ion battery is assembled, the electrolyte will be fully infiltrated with the NTCDA and TNTCDA-NiPc electrode surface, and the SEI film formed during the first charge and discharge will contribute to the large irreversible capacity; secondly, besides being affected by the surface SEI film, the irreversible capacity is strongly affected by solvent co-embedding. Lithium ions can be embedded in electrode materials together with solvent molecules. The solvent co-embedding reaction is determined by the crystallinity and surface structure of electrode materials and the composition of electrolyte used; besides, other factors will also produce irreversible capacity, such as impurities on the surface of electrode materials, irreversible reduction of H_2_O and O_2_, irreversible reduction of functional groups on the inner grain edges of polycrystalline electrode materials, *etc.* Particularly for the NTCDA and TNTCDA-NiPc compound, the molecular structure contains anhydride groups, and a small amount of H_2_O will remain in the assembly process of the battery, which makes the anhydride group hydrolyze to form carboxylic acid, and irreversible lithium carboxylic acid is produced after binding with lithium ions. In addition, a small amount of residual O_2_ will also produce irreversible components such as lithium oxide. All these are important reasons for irreversible capacity. Although the coulomb efficiency of the former three times of the NTCDA electrode and the TNTCDA-NiPc electrode is increasing, it is obvious that the efficiency of the TNTCDA-NiPc electrode is always higher than that of the NTCDA electrode (Fig. 5f). This is mainly because that after the NTCDA is substituted into the phthalocyanine ring, the active groups are effectively dispersed from the molecular point of view. In addition, the sample is a loose porous microstructure. As a result, more active sites are exposed and more unobstructed and faster lithium ion transport channels are available, so that the coulomb efficiency of the TNTCDA-NiPc electrode is higher.

**Fig. 6:**
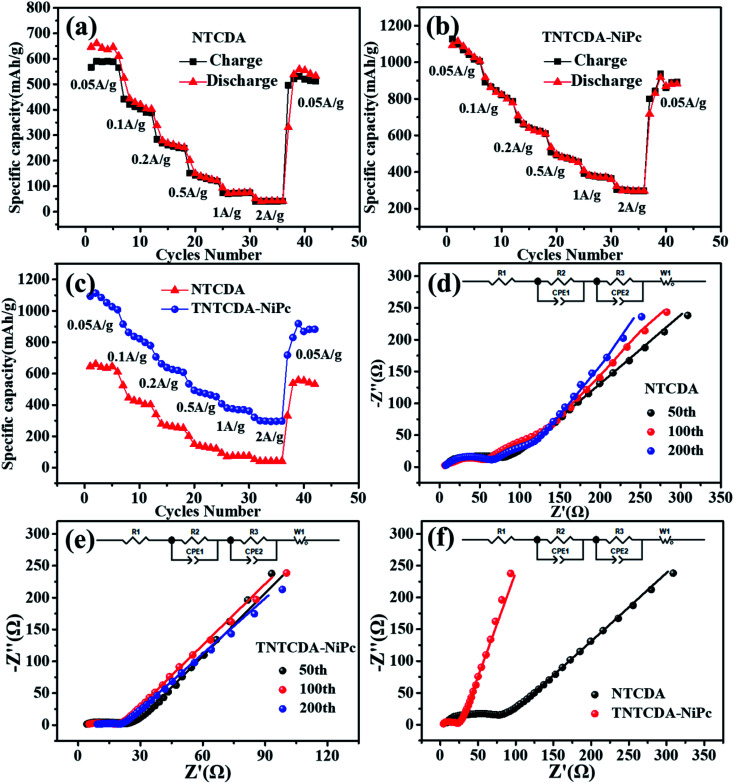
(a–c) Rate performance of the batteries with TNTCDA-NiPc and NTCDA electrodes. (d–f) EIS impedance plots of the TNTCDA-NiPc and NTCDA electrodes.

The rate performance of NTCDA and TNTCDA-NiPc electrodes was studied, as shown in Fig. 6. At current densities of 0.05, 0.1, 0.2, 0.5, 1.0, 2.0 and 0.05 A g^−1^, the discharge specific capacities of the NTCDA electrode are 610.5, 401.9, 251.7, 120.9, 40.5 and 532.1 mA h g^−1^, respectively. However, for the TNTCDA-NiPc electrode, the discharge capacities are 1007.7, 778.6, 607.7, 452.7, 299.3 and 822.8 mA h g^−1^ at the same current densities, which are much higher than those of the NTCDA electrode. At low current densities, the specific capacity of the TNTCDA-NiPc electrode is about twice that of the NTCDA electrode, and at high current density (*e.g.* 2 A g^−1^), the specific capacity is 7 times that of the NTCDA electrode, indicating that the TNTCDA-NiPc electrode has better high rate performance and cycling stability. This is due to the differences in the structure and morphology between the two electrodes; NTCDA is a linear structure with a smooth and flat surface and few Li-ion binding sites on the surface while the TNTCDA-NiPc electrode has a large specific surface area, and more active sites to promote lithium ion transfer and diffusion, which made the TNTCDA-NiPc electrode show an excellent rate performance.

**Fig. 7:**
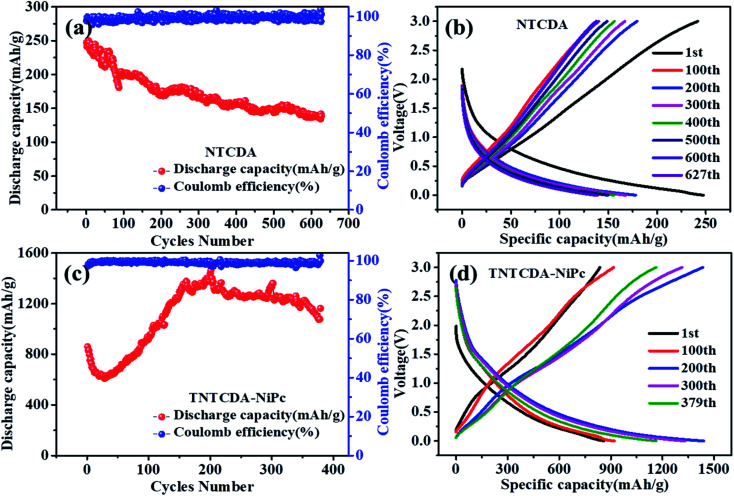
Cycling and charge/discharge curves of the NTCDA electrode (a and b) and TNTCDA-NiPc electrode (c and d).

Conductivity is also one of the important factors affecting the rate performance; therefore, in order to explore the reasons of the capacity difference between the two electrodes, the EIS impedance of NTCDA and TNTCDA-NiPc electrodes through different cycles were investigated as shown in Fig. 6d–f and Table 2. *R*_1_ is the contact impedance and transfer impedance between lithium ions in electrolyte and devices such as a battery shell; *R*_2_ is the interface impedance caused by the solid electrolyte interphase (SEI) film; *R*_3_ is the charge-transfer resistance during an electrochemical reaction; it can be seen that the TNTCDA-NiPc electrode has a decreasing trend in EIS impedance after long cycles, which has a corresponding relationship with the cycle performance of batteries (Fig. 7): the specific capacity decreased gradually at the former 42 times, and increased to a level much higher than the initial discharge capacity from 42 to 150 times, and then maintained a relatively stable specific capacity. Comparing the impedance of the NTCDA and TNTCDA-NiPc electrodes after 50 cycles (Fig. 6f), the interface impedance (*R*_2_) and charge-transfer resistance (*R*_3_) of the TNTCDA-NiPc electrode are much smaller than those of the NTCDA electrode, which proves that the charge-transfer and lithium ion transport abilities are better than those of the NTCDA electrode. The enhancement of charge-transfer ability of the TNTCDA-NiPc electrode is due to the large specific surface area and more exposed active sites brought by its porous structure and molecular dispersion, which leads to lithium ions having faster transmission channels. As a result, the electrochemical performance of the TNTCDA-NiPc electrode is improved.

**Table 2:** List of the EIS fitting parameters of the TNTCDA-NiPc and NTCDA electrodes

Samples	Number	*R*_1_/Ω (error/%)	*R*_2_/Ω (error/%)	*R*_3_/Ω (error/%)
NTCDA	50	7.30 (1.96)	17.66 (17.39)	53.29 (7.32)
	100	3.47 (3.90)	66.59 (1.63)	24.67 (13.81)
	200	5.58 (0.85)	63.22 (0.58)	31.94 (3.95)
TNTCDA-NiPc	50	3.54 (1.44)	11.00 (3.44)	5.78 (7.01)
	100	4.27 (1.45)	8.37 (4.51)	4.20 (8.69)
	200	7.52 (5.30)	7.62 (28.21)	3.47 (43.69)

As shown in Fig. 7, the specific capacity of the NTCDA electrode shows a downward trend; the discharge specific capacity for the first time was 247.4 mA h g^−1^ which was reduced to 138.4 mA h g^−1^ after 627 cycles; the capacity retention is only 55.96%. The results show that the NTCDA compounds show poor cycling stability, which is in good agreement with the dissolution performance in Fig. 2, mainly due to the slow dissolution of NTCDA molecules in the electrolyte and the reduced cycling stability of the batteries; however, after NTCDA was substituted on the Pc ring, the specific capacity of the TNTCDA-NiPc electrode experienced a small decline at the beginning of cycles, then increased rapidly to a stable value. The first discharge capacity is 859.6 mA h g^−1^, and then reduced to 609.7 mA h g^−1^ after 30 cycles. After 379 cycles, the capacity increased to 1162.9 mA h g^−1^; the capacity retention rates increased from 70.9% to 135.3%. The results show that the cycling stability of the TNTCDA-NiPc electrode is greatly improved after substituting NTCDA on the Pc ring due to the improvement of molecular scale dispersion, and the porous structure morphology as well as improved conductivity discussed above. The cycling performance of TNTCDA-NiPc and previously reported similar material electrodes is shown in Table 3, which is much better than those of the reported organic electrodes.

**Table 3:** Cycling performance data of TNTCDA-NiPc and previously reported similar material electrodes

Electrodes	Initial capacity (mA h g^−1^)	200th capacity (mA h g^−1^)	Capacity retention	Ref.
TNTCDA-NiPc	859.6	1438.8	167%	In this work
NTCDA	247.4	170.3	69%	In this work
TN-NiPc	301.4	280.0 (180th)	93%	33
2 (t-CAL-Pc)	190	169 (100th)	89%	34
3 (t-DA-Pc)	274	241	88%	34
Sodium tartrate (ST)	182.3	295.3	162%	35
Sodium pyromellitate (SP)	182.4	246.1	135%	35

In order to illustrate the advantage of TNTCDA-NiPc, a systematic comparison between the performance of TNTCDA-NiPc in this work and that of previously reported similar materials is provided as shown in Table 3. It can be seen that for tetraamino phthalocyanine (TN-NiPc)^33^ and lithium tetracarboxylate phthalocyanine (t-CAL-Pc),^34^ their initial capacity is only 247.4 and 301.4 mA h g^−1^ with faster capacity decay, and for the small molecular carbonyl compounds of sodium carboxylate series (ST and SP), their initial capacity is only 182 mA h g^−1^. However, in this work, after replacing the NTCDA with the PC ring, the dispersion of active unsaturated bonds such as C=O, C=C and C=N is effectively improved from the molecular point of view, which leads to more active sites contributing to capacity, as a result, much higher initial capacity is observed.

**Fig. 8:**
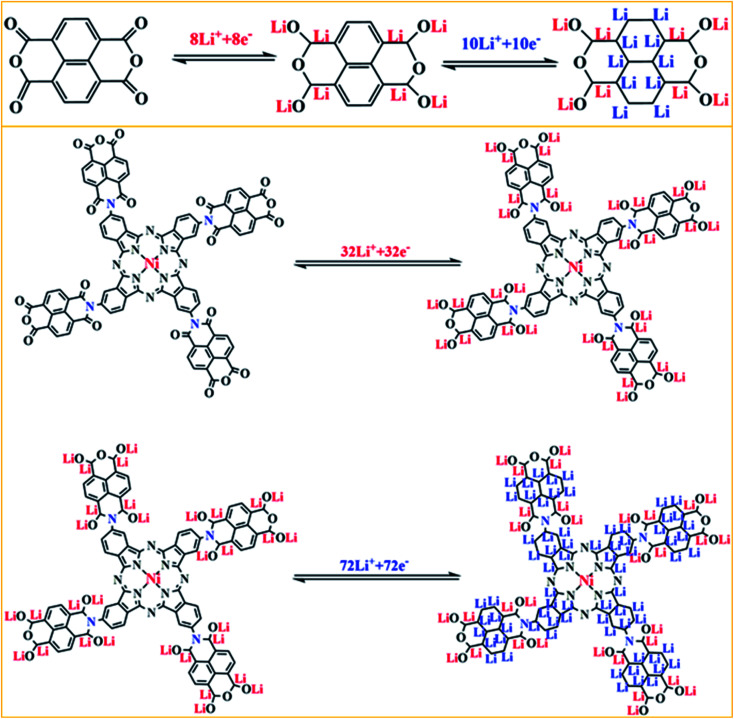
Schematic illustration of the charge/discharge mechanism for the NTCDA and TNTCDA-NiPc electrodes.

The redox peaks of the NTCDA electrode and TNTCDA-NiPc electrode corresponding to different redox reactions, which reflect different electrochemical mechanisms, as shown in Fig. 8. In the NTCDA molecule, the functional groups are the C=C and C=O unsaturated bonds, which correspond to the redox peaks in the CV curves and exhibit a two-step lithium intercalation process. Firstly, 8 Li^+^ combines with the C=O functional group to form O–Li and C–Li bonds, which correspond to the 2.35/1.90 V peak of the CV curves (Fig. 5a); Then C=C on the benzene ring structure of NTCDA reacts with Li^+^, and 10 Li^+^ can be embedded in this step corresponding to the peak at 1.30/0.82 V. These two-step lithium intercalation processes suggest that each NTCDA molecule discharges with 18 Li^+^ to be embedded. However, after NTCDA is substituted to the periphery of the TA-NiPc, the obtained TNTCDA-NiPc molecule contains four NTCDA units. In addition, Pc is a macrocyclic conjugated compound with 18π electrons, which can also contribute to the intercalation of Li-ions. As a result, the C=O, C=C and C=N unsaturated bonds are the factors for electrochemical lithium intercalation in the TNTCDA-NiPc molecules, which also shows a two-step lithium intercalation process corresponding to CV curves (Fig. 5b). The first is the combination of Li^+^ with C=O functional groups in the NTCDA to form O–Li and C–Li bonds; 32 Li^+^ can be embedded in this step corresponding to the 2.4/1.88 V peak of the CV curve; then Li^+^ binding to the C=C in the NTCDA naphthalene ring and the C=C/C=N in the Pc conjugate ring to form C–Li bonds; 10 Li^+^ in each naphthalene ring and 32 Li^+^ on each Pc-ring are embedded. In this step, a total of 72 Li^+^ are embedded corresponding to the peak 0.95/0.76 V in the CV curve. The two-step lithium intercalation processes suggest that each TNTCDA-NiPc molecular discharge can embed 104 Li^+^. It is obvious that TNTCDA-NiPc molecules have more Li-ions binding active sites, which is the decisive factor for its higher specific capacity and excellent electrochemical performance. The layered structure, porous surface and unsaturated functional groups of anode materials are the main factors affecting the specific capacity, and the unsaturated carbon subunits with a conjugate structure can promote the intercalation of Li-ions and contribute greatly to the capacity.^36^

**Fig. 9:**
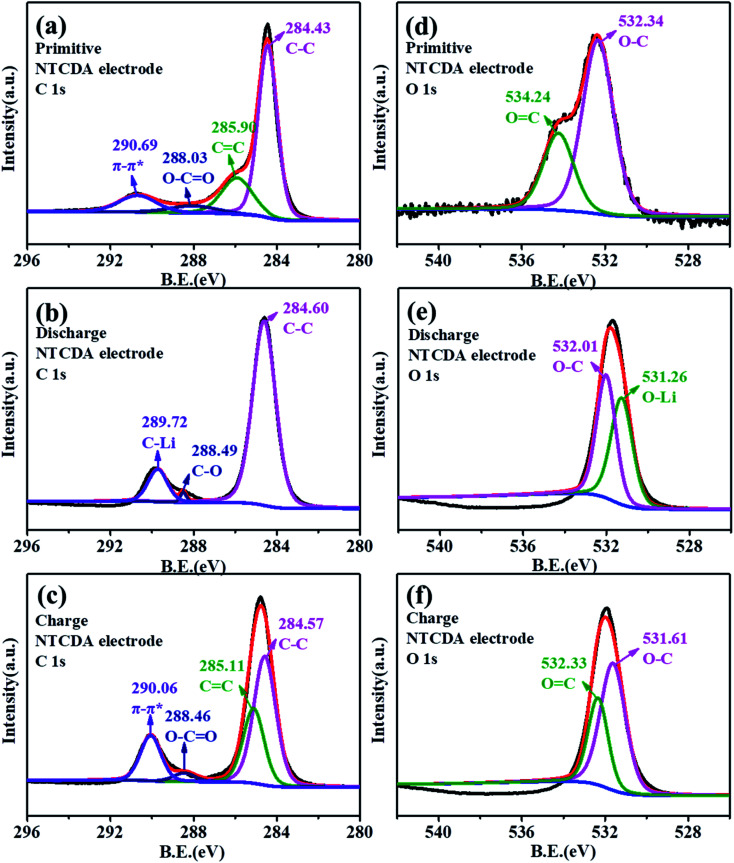
(a–c) C 1s and (d–f) O 1s XPS spectra of the NTCDA electrode. (a and d) Primitive electrode; (b and e) charge electrode; (c and f) discharged electrode.

To further explore the lithium intercalation mechanism of the NTCDA and TNTCDA-NiPc electrodes, the change of valence states of different elements during charge and discharge processes was investigated using XPS spectroscopy. As shown in Fig. 9a, the C 1s spectra of the primitive NTCDA electrode can be deconvoluted to four peaks at 284.4, 285.9, 288.0 and 290.7 eV, which correspond to C–C, C=C and O–C=O structures in the NTCDA electrode, and the NTCDA π–π* conjugate structure, respectively. After the first discharging (Fig. 9b), the Li^+^ combined with O–C=O and C=C to form the C–Li bond. As a result, the peak corresponds to the π–π* group of NTCDA and the C=C group disappeared, and a new peak at 289.7 eV corresponding to the C–Li bond is formed after the combination of Li^+^ with C=C and π–π conjugated groups. Meanwhile, it can be found that the peak at 288.0 eV corresponding to the O–C=O group disappeared which transformed into the C–O group, and the peak at 285.9 eV corresponding to the C=C bond also disappeared and the peak at 284.6 eV corresponding to the C–C group increases significantly. After continuous charging (Fig. 9c), the peak at 290.1 eV represents the NTCDA π–π conjugated structure and the peak at 285.1 eV corresponding to the C=C group reappears, and at the same time, the intensity of the peak at 284.6 eV corresponding to the C–C group decreased significantly. The results of C 1s spectra show the lithium intercalation mechanism of C=C contribution to capacity in the NTCDA molecules.

**Fig. 10:**
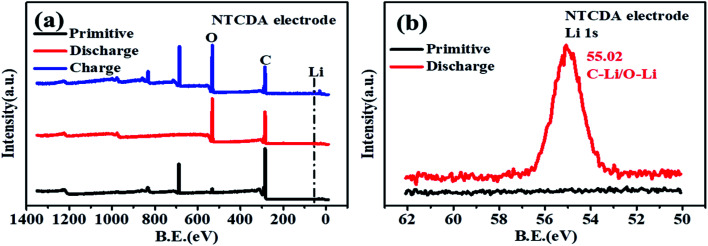
(a) XPS full spectra and (b) Li 1s spectra of the primitive NTCDA electrode before and after discharging.

As shown in Fig. 9d, the primitive O 1s spectrum can be deconvoluted into two peaks at 532.3 and 534.2 eV, corresponding to O–C and O=C groups, respectively. After the first discharging (Fig. 9e), the peak at 534.2 eV corresponding to the O=C group disappeared, and a new peak at 531.3 eV corresponding to the O–Li bond is formed by combining Li^+^ with the O=C group, with the intensity of the O–C peak decreasing significantly. After recharging (Fig. 9f), the peak at 532.3 eV corresponding to the O=C group was observed. The results also indicate that the C=O bond on the NTCDA structure also contributes to the capacity. The changes of C 1s and O 1s spectra show the lithium-intercalation mechanism of charge–discharge process as shown in Fig. 10a. Meanwhile, the Li 1s spectra of the NTCDA electrode before and after discharging (Fig. 10b) are also investigated. The primitive electrode did not show Li elements, but the peak of Li at 55.0 eV is observed after discharging, indicating that Li-ions are embedded in the NTCDA electrode during the discharge process.

**Fig. 11:**
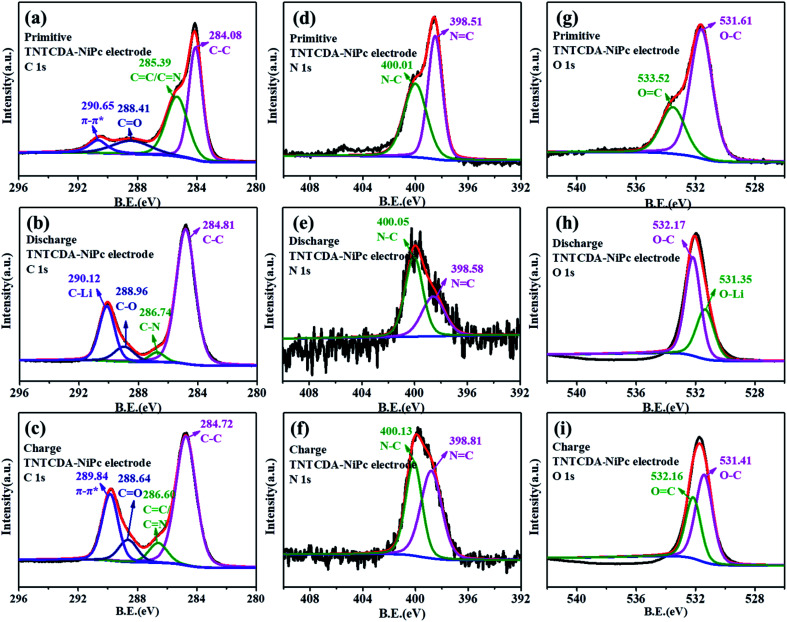
(a–c) C 1s, (d–f) N 1s and (g–i) O 1s XPS spectra of the TNTCDA-NiPc electrode. (a, d and g) Primitive electrode; (b, e and h) charge electrode; (c, f and i) discharged electrode.

The XPS spectra of the TNTCDA-NiPc electrode are also investigated to evaluate its lithium-intercalation mechanism. As shown in Fig. 11a, the C 1s spectrum of the primitive TNTCDA-NiPc electrode can be deconvoluted into four peaks at 284.1, 285.4, 288.4 and 290.7 eV, respectively corresponding to the C–C, C=C/C=N, and C=O groups in the TNTCDA-NiPc molecules, and the π–π* conjugated structure of the Pc benzene ring. Similarly, the peak at 290.7 eV corresponding to the π–π* group disappeared after the first discharging (Fig. 11b), and a new peak at 289.1 eV corresponding to the C–Li bond is formed after lithium intercalation, indicating that the Pc conjugated structure is involved in the process of lithium intercalation. In addition, the peaks corresponding to the C=N and C=O groups disappeared, and the C–N peak at 286.7 eV and the C–O peak at 289.0 eV are observed. At the same time, the intensity of the peak at 284.8 eV corresponding to the C–C group was enhanced significantly, fully revealing the lithium intercalation process of C=C, C=O and C=N bonds. After recharging (Fig. 11c), the peak at 289.8 eV corresponding to the π–π* group, the peak at 288.6 eV corresponding to the C=O group and the peak at 286.6 eV corresponding to the C=O/C=N groups reappeared, fully revealing the delithiation process of C=C, C=O and C=N bonds. The lithium intercalation mechanism of C=N bonds can also be confirmed from N 1s spectra. The N 1s spectrum of the primitive electrode can be deconvoluted into two peaks at 398.5 and 400.0 eV (Fig. 11d), corresponding to the N=C and N–C groups in TNTCDA-NiPc molecules, respectively. After discharging (Fig. 11e), the intensity of the peak at 398.6 eV corresponding to N=C decreased obviously, and the peak at 400.0 eV corresponding to N–C increased significantly, revealing the lithium intercalation process of C=N bonds in the TNTCDA-NiPc molecules. After recharging (Fig. 11f), the intensity of the peak at 398.6 eV corresponding to N=C increased obviously, revealing the delithiation process of C=N bonds in the TNTCDA-NiPc molecules.

**Fig. 12:**
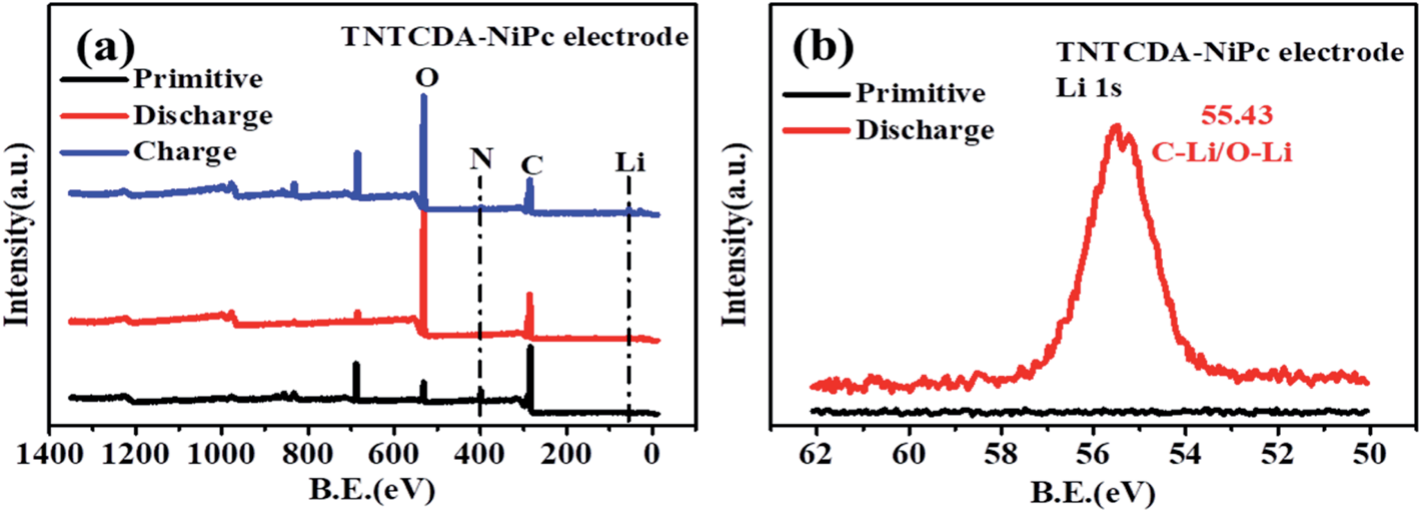
(a) XPS survey spectra and (b) Li 1s spectra of the primitive TNTCDA-NiPc electrode before and after discharge.

The primitive O 1s spectrum can be divided into two peaks at 531.6 and 533.5 eV, corresponding to O–C and O=C groups in the TNTCDA-NiPc molecules respectively (Fig. 11g). After the first discharging (Fig. 11h), the peak at 533.5 eV corresponding to the O=C group disappeared, and a new peak at 531.4 eV correspondingt to the O–Li bond is formed after the lithiation reaction of Li^+^ with the O=C group, with the intensity of the O–C peak decreasing obviously. After being charged (Fig. 11i), the peak at 532.2 eV corresponding to the O=C group is observed again. The results fully revealed the mechanism of lithium intercalation and de-lithiation of C=O bonds in the TNTCDA-NiPc molecules. The changes of C 1s, O 1s and N 1s spectra show the lithium-intercalation mechanism of the charge–discharge process as shown in Fig. 12a. Meanwhile, the Li 1s spectrum of the NTCDA electrode before and after discharging is also investigated (Fig. 12b); the primitive electrode did not show Li elements, but the peak of Li at 55.4 eV is observed after discharging, which indicated that Li-ions are embedded in the TNTCDA-NiPc electrode during the discharging process.

The XPS elemental valence analysis of NTCDA and TNTCDA-NiPc electrodes shows that the C=O group and C=C conjugated structure are the active functional groups and contribute greatly to the capacity, and have good reversibility. In addition, the TNTCDA-NiPc electrode also has a Pc macrocyclic conjugated structure contributing to capacity, which is also one of the factors for its capacity being higher than that of the NTCDA electrode.

**Fig. 13:**
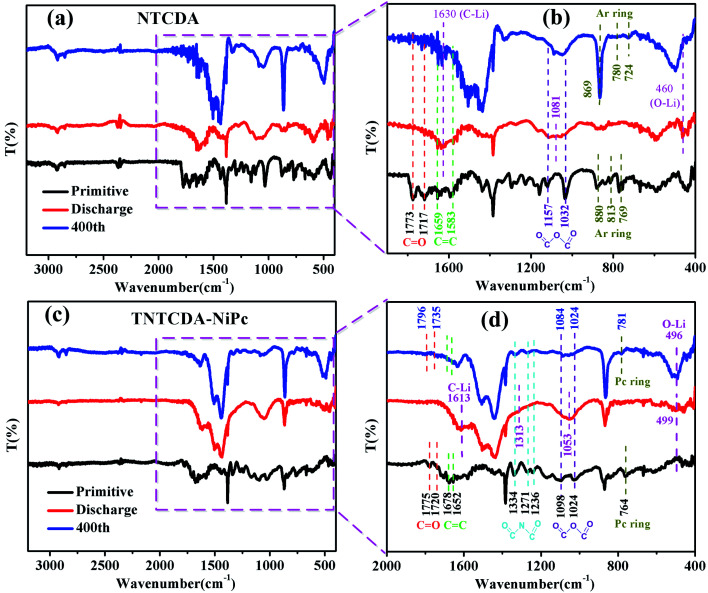
FTIR spectra of electrodes before and after charge/discharge (a) NTCDA; (b) TNTCDA-NiPc.

In order to understand the structure change of the compound during charge and discharge more clearly, the FTIR spectra of NTCDA and TNTCDA-NiPc electrodes before and after charge/discharge are investigated. As shown in Fig. 13a, for the NTCDA primitive electrode, the peaks at 1773 and 1717 cm^−1^ are attributed to the tensile vibration of the C=O bonds, and the peaks at 1659 and 1583 cm^−1^ are attributed to the tensile vibration of the C=O bonds, respectively. After the first discharging, the Li^+^ combined with C=O and C=C bonds to form C–Li and O–Li bonds, respectively. As a result, the peaks at 1773 and 1717 cm^−1^ attributed to the tensile vibration of the C=O bonds disappeared, and two new peaks at 1630 cm^−1^ corresponding to the C–Li bond and at 460 cm^−1^ corresponding to the O–Li bond appeared (Fig. 13b). Similarly, the peaks at 1157 and 1032 cm^−1^ can be ascribed to the tensile vibration of the O=C–O–C=O bond, and the peaks from 880 to 769 cm^−1^ can be ascribed to the bending vibration of the Ar ring. After discharge, the intensity of the peaks corresponding to the tensile vibration of the O=C–O–C=O bond and the bending vibration of the Ar ring are greatly weakened, and at the same time, a new peak at 1081 cm^−1^ corresponding to the tensile vibration of the –C–O–C– bond originated. After charging at 400 cycles, partial reversible lithium deintercalation causes unsaturated double bonds to re-form. As a result, those peaks corresponding to the C=O, C=C, O=C–C–C=O, and Ar ring appeared again or are enhanced. The results further indicate that the unsaturated C=O and C=C bonds are involved in the reaction of lithium intercalation.

For the TNTCDA-NiPc primitive electrode (Fig. 13c and d), the peaks at 1775 and 1720 cm^−1^ are attributed to the tensile vibration of the C=O bond, and the peaks at 1678 and 1652 cm^−1^ are ascribed to the tensile vibration of the C=C bond. After discharge, it’s clearly seen that the peaks of the tensile vibration of the C=O and C=C bonds disappeared, and new peaks at 1613 cm^−1^ corresponding to the C–Li bond and at 499 cm^−1^ corresponding to the O–Li bond are observed. In addition, the series peaks from 1334 to 1236 cm^−1^ correspond to the tensile vibration of the O=C–N–C=O bond, and the peaks from 1334 to 1236 cm^−1^ corresponding to the tensile vibration of the O=C–O–C=O bond also disappeared after discharge, and new peaks at 1313 cm^−1^ corresponding to the –C–N–C– bond and 1053 cm^−1^ corresponding to the –C–O–C– bond are observed. Furthermore, the peak at 764 cm^−1^ corresponding to the bending vibration of the Pc ring also disappeared after discharge. However, after charge at 400 cycles, partial reversible lithium deintercalation causes unsaturated double bonds to re-form. As a result, those disappeared peaks of C=O, C=C, O=C–C–C=O and the Ar ring reappeared. The results further indicate that the unsaturated C=O C=C and C=N bonds on the TNTCDA-NiPc molecules are involved in the reaction of lithium intercalation and contribution to the capacity.

**Fig. 14:**
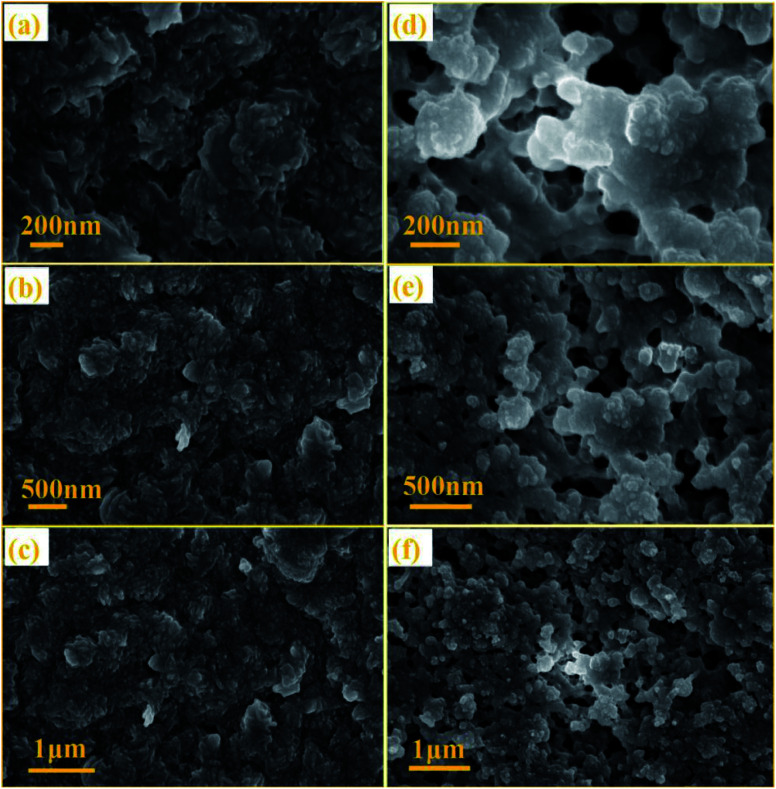
SEM images of NTCDA electrodes (a–c) and TNTCDA-NiPc electrodes (d–f) after 400 cycles.

The NTCDA and TNTCDA-NiPc electrodes have many differences in the molecular structure, micro-morphology and lithium intercalation state, in order to find out the difference between the two electrode materials during the charge–discharge process; SEM images were recorded on the electrodes after cycling 400 times, as shown in Fig. 14. It can be seen that the molecular particles in the NTCDA electrode after 400 cycles are very dense (Fig. 14a–c). The NTCDA particles are closely agglomerated together and become the whole block structure of large particles, which makes the exposed active sites limited, which is very unfavorable to the transfer of Li-ions and the presentation of capacity. This is the main factor for the decrease of the specific capacity of the NTCDA electrode after long cycles. However, as shown in Fig. 14d–f, the TNTCDA-NiPc electrode shows a loose porous microstructure with a large specific surface area; there are many tiny pores between particles that expose more active sites, which are more conducive to lithium ion transport and the capacity contribution of active groups.

**Fig. 15:**
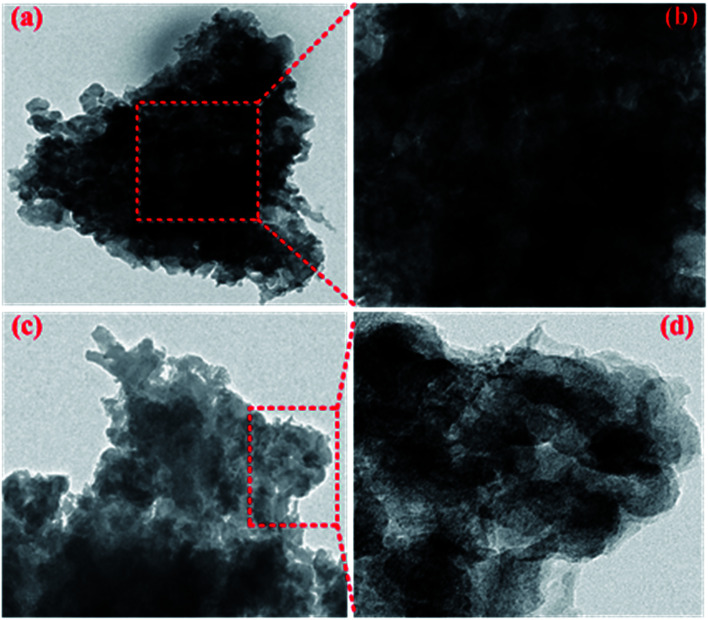
TEM images of NTCDA electrodes (a and b) and TNTCDA-NiPc electrodes (c and d) after 400 cycles.

This result can be further concluded from the TEM images of the electrode sheets after 400 cycles. NTCDA molecules are more likely to agglomerate together to form a dense electrode material (Fig. 15a and b), which results in low utilization of active sites and the capacity of internal active groups can not be effectively brought out. In addition, NTCDA molecules can slowly dissolve in the electrolyte, which results in its low capacity and poor cycling stability. However, after NTCDA is substituted on the Pc rings, the NTCDA units and the Pc units are effectively dispersed on the molecular scale. At the same time, the TNTCDA-Pc electrode shows a porous and loose microstructure (Fig. 15c and d), which exposes more active sites and improves the conductivity effectively, which is more conducive to lithium ion transport and the capacity contribution of active substances, and would provide a new idea for designing molecular models of high performance organic electrode materials.

## 4 Conclusions

In this work, large-volume branched phthalocyanine TNTCDA-NiPc was synthesized by a simple template hydrothermal synthesis method; the small molecule oxygen-containing organic compound NTCDA was grafted onto a structurally stable Pc-ring; the obtained TNTCDA-NiPc compound displays non-solubility in electrolyte and a porous and loose microstructure. As a result, it shows improved electrochemical performance when used as an anode material for lithium ion batteries compared with the NTCDA electrode. Firstly, NTCDA is substituted on the Pc-ring with a stable structure, which reduces its solubility significantly and facilitates it stable existence in the electrolyte. Secondly, the porous molecular structure after substitution is very favorable for lithium ion transport and improves the diffusion and transfer rate of lithium ions. Thirdly, the conjugated structure of Pc macrocycles promotes charge transfer and improves conductivity, which makes the TNTCDA-NiPc electrode present a faster redox rate and more stability at high current charge/discharge. Moreover, after NTCDA substitution on the Pc-ring, the NTCDA units and Pc units are more dispersed at the molecular level, which is conducive to the utilization of active sites. These are the reasons that the TNTCDA-NiPc electrode displays higher capacity, better cycle stability and rate capability than the NTCDA electrode.

## Conflicts of interest

There are no conflicts to declare.

## Supplementary Material
